# Team Creative Environment as a Mediator Between CWX and R&D Team Performance and Moderating Boundary Conditions

**DOI:** 10.1007/s10869-017-9495-8

**Published:** 2017-04-05

**Authors:** Mar Bornay-Barrachina, Inés Herrero

**Affiliations:** Department of Management and Marketing, Universidad Pablo de Olavide, Carretera de Utrera, Km 1, 41013 Seville, Spain

**Keywords:** Co-worker exchange relationships, Creative team environment, Team performance, Research output, R&D teams

## Abstract

**Purpose:**

The purpose of this study was to investigate how high-quality dyadic co-worker relationships (CWXs) favour or hinder team performance. Specifically, we examine the role played by CWX, team creative environment, job complexity and task interdependence to achieve higher levels of team performance.

**Design/Methodology/Approach:**

We analyse data from 410 individuals belonging to 81 R&D teams in technology sciences to examine the quality of the dyadic relationships between team members under the same supervisor (co-workers) and team performance measured by the number of publications as their research output.

**Findings:**

Higher levels of team average CWX relationships are positively related to the establishment of a favourable creative team environment, ending into higher levels of team performance. Specifically, the role played by team average CWX in such relationship is stronger when job complexity and task interdependence are also high.

**Implications:**

Team’s output not only depends on the leader and his/her relationships with subordinates but also on quality relationships among team members. CWXs contribute to creative team environments, but they are essential where jobs are complex and tasks are highly dependent.

**Originality/Value:**

This study provides evidence of the important role played by CWXs in determining a creative environment, irrespective of their leaders. Previous research has provided information about how leader’s role affects team outcomes, but the role of dyadic co-worker relationships in a team remains still relatively unknown. Considering job complexity and task interdependence variables, the study provides with a better understanding about how and when high-quality CWXs should be promoted to achieve higher team performance.

## Introduction

R&D team performance has been related with creative and innovative results (Gu et al. 2013; Kratzer et al. 2006). To explain individual and group creative behaviour, research has focused on personal and/or relational factors (Bakker et al. 2013; Fliaster and Schloderer 2010; Rickards and Moger 2000; Sijbom et al. 2015), suggesting that team environments favour team innovation (Peltokorpi and Hasu 2014).

Team environment can be understood as a group of stimuli that can be perceived and interpreted. The interaction and exchanges with others can influence on the environment’s perceptions. In this sense, relationships within teams can promote learning, adaptive performance and innovation (Aalbers et al. 2014), as they provide support for innovation and shared vision. However, the studies on work relationships usually highlight high-quality dyadic relationships between leader and subordinate (leader-member exchange, LMX) (Anand et al. 2010; Chiaburu et al. 2013; Liao et al. 2010)—that is, relationships of trust, mutual obligation and respect (Graen and Uhl-Bien 1995)—whereas studies on dyadic co-worker relationships and its relation to team outcomes are scarce. While the varying quality of leader and subordinate relationships may determine whether or not subordinates perceive their environment as a safe and comfortable place to develop and exchange ideas (Amabile et al. 1996; Tierney 1999), such perceptions also reflect the interactions between team members (Banks et al. 2014; Love and Dustin 2014; Woodman et al. 1993). Chiaburu and Harrison (2008) found evidence that co-worker actions predict the perceptual, attitudinal and behavioural outcomes of their colleagues even net of the influence of the direct leaders. Still, Omilion-Hodges and Baker (2013) state, “Decades of literature seek to explain how leaders affect individual, group and organizational level outcomes, yet in privileging the leader, the role that peer relationships play in overall group functioning remains relatively unknown” (2013, p. 937; for a similar call for research, see Tse and Dasborough 2008, p. 213).

Literature examining how team member relationships can affect creativity and innovation at work (Dunegan et al. 1992; Seers et al. 1995) has usually considered the interactions between individuals and the team as a whole in such a manner that collective perceptions are considered (TMX; Seers et al. 1995; and WGX; Dunegan et al. 1992). However, literature on the quality of dyadic relationships (e.g. Olsson et al. 2012; Omilion-Hodges and Baker 2013) has shown that relations at the dyad-level affect the individual perception of the work environment, and despite that co-worker support and influence within dyads have been shown to be critical in obtaining citizenship behaviour, job satisfaction, organizational commitment or effectiveness (Chiaburu and Harrison 2008; Raabe and Beehr 2003), little is known about how the quality of dyadic co-worker relationships affects team performance. The dyadic relationship between peers reporting to the same manager and belonging to the same team (co-worker exchange relationship, hereafter CWX) (Sherony and Green 2002) can be measured in terms of the respect, trust and mutual obligation between them (Jacobs 1970; Jones 2001). Differences in the quality of these relationships may influence the individual perception about the environment and its consideration as appropriated to develop ideas.

However, the complexity and the interdependence of tasks performed within a team can also affect interactions and the frequency of communication among team members (Man and Lam 2003). In this study, we analyse the direct and indirect effects of high-quality co-worker relationships on team performance, measured in terms of research output, taking into account the potential influence of job complexity and interdependence task on the perception of a creative team environment. We examine R&D teams, which are often multi-functional and multi-disciplinary, with the flexibility and the ability to communicate and execute quickly. These teams tend to be highly self-organized; therefore, the role played by the quality of team members’ relations could be relevant for the team to perform properly (Stoker et al. 2001:1141).

This paper aims to make several contributions, the first of which is to synthesize, from the extensive literature on CWX and LMX, evidence of the important role played by the quality of dyadic relations within a team in determining a creative environment, irrespective of their leaders. Second, our study sheds light on creativity processes by showing how relationships at work combine with job complexity and task interdependence to generate an appropriate environment for creativity. Third, we test the team environment for creativity as a mediator between average team quality co-worker relationships and team performance, showing that relationships among peers reporting to the same leader can foster innovative output. Fourth, we also contribute to literature on R&D teams, sorely under-researched despite the importance of creativity. Last, we use objective measures for research output (Olsson et al. 2012) and thus avoid the common source bias found in studies that use self-reported measures (e.g. Kratzer et al. 2006).

## Theoretical Background and Hypotheses

### Dyad Co-worker Relationships and the Creative Team Environment

A creative team environment can be defined as a work environment that encourages and supports creativity among its members (Gilson et al. 2005), and work environment can be defined as a group of stimuli that are perceived and interpreted (Kopelman et al. 1990) where interaction and exchange with others influence that perception (Salancik and Pfeffer 1978). Therefore, a creative team environment can be understood as a group of stimuli encouraging and supporting creativity where interaction and exchange with others can influence and determine such a perception. Although extensive research shows leaders as key players in determining climate and work environments for creativity and innovation (Amabile et al. 1996; Eisenbeiss et al. 2008; Tierney 1999; West 1990), and leaders are crucial for determining the direction and frequency of interactions, interactions among team members do not necessarily depend solely on their leader. For example, a meta-analysis by Chiaburu and Harrison (2008) found that in jobs with high-intensity social requirements, co-workers influenced individual and organizational outcomes more than leaders did: “because of specific interpersonal components of particular tasks and positions (e.g., need to cooperate), co-workers matter more for their colleagues’ roles, attitudes, withdrawal and effectiveness in these settings than for jobs with reduced social intensity” (2008:1095).

The success of a relationship is based on the exchange of benefits, either intrinsic or extrinsic, as well as on normative obligations. At work, social environments are frequently defined by the co-workers (Clay and Olitt 2012). Just as with LMXs, the high quality of CWX relationships translates into high dyadic levels of trust, mutual obligation and reciprocity (Omilion-Hodges and Baker 2013; Raabe and Beehr 2003; Wikaningrum 2007), and variations in quality may affect peers’ perceptions of their environment (Bommer et al. 2003; Jordan et al. 2002).

CWXs have been studied in relation to work attitudes (Sherony and Green 2002; Wikaningrum 2007), burnout and work motivation (Fernet et al. 2010) and co-worker resources (Omilion-Hodges and Baker 2013), and it has also shown that a high quality of co-worker relations could favour creativity in much the same way as leader-member exchange (for reviews, see Mumford et al. 2002; Tierney 2008).

Love and Dustin (2014) noted that a supportive environment encourages change-oriented behaviour and fosters generating and implementing new ideas. Members need to feel comfortable enough to take risks, exchange information and encourage the search for new ideas and solutions (Amabile et al. 1996). Friendly and supportive behaviour allows followers to share knowledge and resources (Graen and Uhl-Bien 1995; Jones 2001), a process that is especially important when new tasks are to be undertaken. A meta-analysis on team innovation by Hülsheger et al. (2009) showed that high-quality internal communication among team members represents a key factor on team innovation.

When co-workers achieve high levels of trust, respect and mutual obligation, they can interact more frequently and more effectively, sharing information and feeling comfortable enough to generate new ideas (Baer and Oldham 2006). It then becomes easier to promote a creative team environment (Amabile et al. 1996). Such an environment fosters positive feedback, cooperation and willingness to help (Kluger and DeNisi 1996). Zhou and George (2001) argue that co-workers play a crucial role in the perception of creativity and innovation through constant feedback (Agrell and Gustafson 1994). Albrecht and Hall (1991) observed that suggesting new ideas is risky, since changes to a previously established order subject innovators to scrutiny from the rest of the members in an organization; high-quality relationships with team members reduce this risk.

With higher averages for the dyads within a team relates to a more positive perception of creative team environment. Given that perception of the environment is the result of socially constructed interactions (Schneider 1973), much of what team members perceive is dependent on the attitudes and perceptions of the other team members. In that way, we expect that:

- Hypothesis 1. Team average of CWXs will be positively related to creative team environment.

### The Creative Team Environment and Team Performance

At the end of the 1980s and during the 1990s, researchers including Woodman et al. (1993) and Amabile et al. (1996) showed that perceived work environment influences both the level and the frequency of creative behaviour, either positively or negatively; factors include the sense of control and autonomy, the perception of what constitutes a challenge or urgency and the support of supervisors and team members. Creativity may be encouraged by interaction with others and willingness to help (Diliello et al. 2011; Hooper and Martin 2008). For instance, Baer and Oldham (2006) showed a correlation between support for creativity received from supervisors and co-workers and openness to experiment. Hackman (1992) points out that, in some cases, new ideas may be either rejected or ignored, while in others they appear attractive and receive practical support. Sharing these ideas with the rest of the group may increase opportunities to produce new ideas, although it also implies that team members must consider the ideas of others (Paulus 2000). Zhou and George (2001) showed that feedback and helpful and supportive behaviour from co-workers were crucial to employees’ creativity levels. Creativity requires examining old problems from different perspectives, combining unrelated processes and products to make something new. According to Madjar (2008), emotional and informational support from co-workers creates a pool of resources and enhances creative performance. Barczak et al. (2010) mentioned team trust and collaborative culture as antecedents of team creativity. De Dreu and West (2001) showed that team members need to share knowledge and work in order to transform innovative ideas into feasible processes, products or services. High-quality relationships among colleagues who cooperate, share feedback and provide mutual support may improve with the frequency and quality of communication. Recently, a meta-analysis by Chiaburu et al. (2013) showed that change-oriented citizenship depends on support received from employees’ social context, that is to say, from leaders, co-workers and organizations. In sum, an environment that facilitates intense interactions among team members, constant feedback and a comfortable and safe environment for the presentation of new ideas is likely to foster creative output (Zhou and George 2001).

In parallel, research on team innovation (Anderson and West 1998; Keller 2006; West 1990, 2002; West et al. 2003) has examined team processes as antecedents of team innovation (e.g. Rousseau et al. 2013; Seyr and Vollmer 2014). West (1990) suggests that support for innovation and climate for excellence are key factors in teams’ ability to innovate. Support for innovation reflects cooperative and collaborative behaviour, while climate seems to reflect team members’ commitment to high-quality standards and clear performance criteria within the team (West 1990). Anderson and West (1998) developed a measure of climate for workgroup innovation that included vision, participative safety, task orientation and support for innovation (see also Mathisen et al. 2006; Ragazzoni, Baiardi, Zotti, Anderson, & West 2002). This type of environment generates safety during interactions with other members (Paulus 2000; Edmonson 1996). Gu et al. (2013) believe that managers seeking to promote R&D innovation in teams need to cultivate a work environment with healthy interactions that facilitates psychological safety; this point is echoed by Edmonson (1999) and West (1990). Edmonson (1996), who analysed differences in team management in a hospital, observed that some group’s members discussed their medication mistakes as well as new ways of preventing them, whereas other members concealed this type of information and preferred not to be involved in such discussions. All in all, research shows that an environment which promotes the exchange of information, tolerance of errors and psychological safety clearly encourages creative and innovative results (Gu et al. 2013):

- Hypothesis 2: Creative team environment will be positively related to team performance.

Taken together, hypotheses 1 and 2 allow us to consider the mediating role of the team creative environment in the relationship between team average co-worker relations and team performance (Amabile et al. 1996; Hunter et al. 2007; Mathieu et al. 2008). Stoker et al. (2001) stated that team characteristics like cohesion and organizational citizenship mediated between leadership and innovation for R&D teams. Wong et al. (2009) used mediator variables of potency and climate to document empirically how relationships facilitated team innovation. Eisenbeiss et al. (2008) found that support of innovation—understood as the extent to which team members behave supportively to facilitate the development and implementation of new ideas within the team—mediated the relationship between transformational leadership and team innovation. Finally, more recently, Rousseau et al. (2013) showed that mediation occurred through team goal commitment between team coaching and team innovation. Like support for innovation, a creative environment favours a flow of supportive behaviour on the part of team members. Therefore,

- Hypothesis 3: Creative team environment will mediate the relationship between team average of CWXs and team performance.

### The Moderator Role of Job Complexity and Task Interdependence

Task development determines who will be part of the group, which roles members will undertake, how they will have to work as a team and which processes will be performed individually or collectively (Richter et al. 2011). This implies that the task is directly related to varying degrees of creativity and innovation. In general terms, creative tasks are ill-defined, complex and challenging, characterized by high levels of autonomy and by variety in abilities needed, identity and feedback. These types of task, in turn, usually strengthen intrinsic motivation, benefiting creativity (Amabile 1988).

Perceived job complexity reflects a team member’s belief that different levels of variety, significance, identity, feedback and autonomy in tasks make the work interesting and challenging (Hackman and Oldham 1980). We propose that job complexity moderates the relationship between co-worker relationships and team creative environment for two reasons. First, the more complex the tasks, the more motivated, satisfied and productive employees feel. There are several studies focusing on the relationship between work complexity and creative responses from employees. Hatcher et al. (1989) found that work complexity correlated with the number of new ideas generated by employees. Oldham and Cummings (1996) concluded that employees with personal creative skills are drawn to complex tasks, as these provide them with the tools to develop their potential, whereas simple and automatic tasks may inhibit their potential.

Second, complex and difficult tasks result in higher intrinsic motivation; task complexity may mean that more feedback is necessary between colleagues (Van der Vegt et al. 2000) because of the need for further information and knowledge. Complex tasks generally require more interaction within the group, higher coordination and interdependence (Wood 1986). Complex tasks can therefore be characterized as being ambiguous, ill-structured and complicated, which means that group members need cooperation and coordination to carry them out successfully. Thus, the more complex the tasks, the greater the need for mutual support and understanding among team members. In contrast, simple tasks require only established procedures, rendering discussion and coordination unnecessary (Man and Lam 2003). Recently, Wang et al. (2014) found that the effects of shared leadership were stronger when the work of team members was more complex, as team members needed multiple co-workers to be involved in information and perspective sharing. In sum, high levels of average CWXs in a team interact with high job complexity to increase motivation and need for interaction and support, sharpening team members’ perception of the team creative environment.

However, when team members cannot work independently to each other when completing a task, holding good relationships among them becomes vital to create an appropriated environment. The interdependence of the task, understood as the extent to which each team member needs the other members to develop and complete his/her tasks (Gilson and Shalley 2004), implies the need for good interactions among team members; however, the level of interdependence necessary to carry out certain tasks may differ, depending on how the tasks have been designed (Wageman 1995).

Johnson and Johnson (1989) called the attention to the benefits of designing task with high interdependence, citing its positive effects on learning, achievement, cognitive complexity of thought and relationships. In the same line, other authors (Shea and Guzzo 1989; Wageman 1995) have identified interdependence of the task as a potential key element for group effectiveness. High levels of interdependence of the tasks increase the level of coordination, communication, support and information sharing in individualist tasks (Johnson 1973), thus influencing in the levels of social interaction. Interdependence of the task should allow interaction of members, risk taking and open discussions; therefore, consequently, the levels of cooperation and collaboration should be high, promoting high climate for creativity and innovation. However, for all this happening, the team needs to hold strong and good relationships among its members. Furthermore, the higher the job complexity, the higher the need for cooperation and collaboration and the need to work as a team to create a suitable environment for creativity.

For these reasons, we argue that in situations of high job complexity, high levels of interdependence of the tasks and of CWX are needed in order to develop a suitable creative environment. On the opposite side, if the job is less complex or interdependence of task is low, CWX plays a much weaker role in the development of a climate for creativity. All the previous arguments lead us to the following hypothesis:

- Hypothesis 4: There is a three-way interaction among job complexity task interdependence and team average of CWXs on creative team environment, in such a manner that the relationship between team average of CWXs and creative team environment will be reinforced when job complexity and task interdependence are both high.

**Fig. 1:**
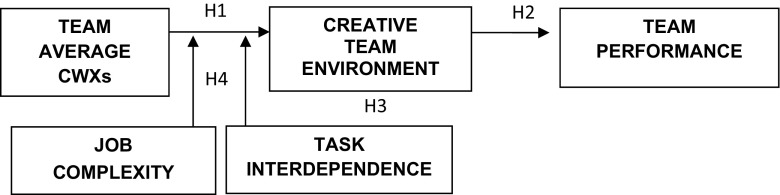
Theoretical assumptions and hypotheses

Figure 1 shows all the theoretical relationships and hypotheses.

## Methods

### Sample and Procedure

Various researchers have used R&D teams to study creativity and innovation (e.g. Chi et al. 2009; Shin and Zhou 2007; Smith et al. 2005). The advantages of R&D teams include their ability to bring together scientists and engineers from different disciplines and the focus on responsibility for integration and task completion. Our sample was composed of R&D teams in the field of physical technology sciences belonging to a consortium of research organizations from a European country (for anonymity reasons, we have omitted for the moment the country of origin). We used the research institution’s webpage to generate a list of the main research lines and associated projects, obtaining the name and contact details for each project head. We held telephone conversations with all team leaders to obtain more information about the structure of these teams and their availability for participation in this study. Team members are mostly university professors, scientists, engineers and technicians.

In the first step, we mail out a questionnaire collecting information about our variables: co-worker exchange relationships, creative team environment, job complexity and task interdependence. We also collected information about team performance 2 years later as the second step. The response rate was 38% for team members and 29.9% for leaders. We only considered those questionnaires for which we had both leaders’ responses and responses from at least 75% of the team members. We accounted a total of 410 questionnaires, constituting a final sample of 81 teams. The teams ranged in size from 3 to 17 members (leaders included), with an average size of 5.64 (sd = 2.68). The team members had worked with their research group on average for 40 months (sd = 17.94).

### Measures

*CWX.* We used the CWX scale from Sherony and Green (2002), who slightly modified LMX-7 (Graen and Uhl-Bien 1995), to capture the quality dyadic relationship among co-workers in terms of respect, trust and mutual obligation. Team members answered a complete scale of six items for each of their co-workers in a team. Crombach alpha indicated a good internal consistency, being equal to 0.88.

#### Creative Team Environment

We measured creative team environment using Gilson et al.’s (2005) scale composed of three different items. Members were asked whether the team as a whole is encouraged to introduce changes or to try new things even when these are not useful. Crombach alpha was 0.8.

#### Job Complexity

To measure job complexity, we used Dean and Snell’s (1991) scale composed of three items whose aim is to unravel mental processes applied in specific tasks, such as problem solving, discretion and technical knowledge application. We tested for reliability and found that Cronbach’s alpha was equal to 0.64.

#### Task Interdependence

We used the three-item scale from Gilson and Shalley (2004) that measures the degree that each team member needs the rest of the team to complete his or her task. Cronbach’s alpha indicated a good internal consistency, being equal to 0.88.

#### Team Performance

The unit of analysis in this study is the scientific research team. When we interviewed team leaders by telephone, 96% of the interviewees agreed with Nederhof (2006), Lexchin et al. (2003) and Payne (1987, 1990) that innovative and original results could be measured by the number of published papers. After talking to team leaders about published papers, we used the ISI Web of Science databases the research institutions’ webpages to verify and complete lists. The data for these measures were then gathered from the annual scientific reports on the websites 2 years after survey data collection (end of 2012 questionnaire data collection period; beginning of 2015 team performance data collection). All measures were collected for a 5-year period starting 1 year into the team’s lifecycle. The number of published papers per team ranged from 0 to 25, with a mean of 6.83 (sd = 4.18). We originally thought about considering the number of new products developed by the team, but it was zero in most cases being the average amount of new products less than 1, the reason for which we discarded it as a variable to our analysis.

#### Control Variables

Simonton (1988) demonstrated that scientific productivity is related to experience and length of stay within the field. We measured team member *tenure* as the number of months working within the team. Cohen and Bailey (1997) have shown different types of relationship between *team size* and effectiveness, with curvilinear or direct effects; therefore, both tenure and team size have been included as control variables affecting team creative environment and team performance. Leader-Member Exchange (LMX), understood as the quality of the relationship that the leader holds with each of the subordinate (Graen and Uhl-Bien 1995), has also been acknowledged to affect how the team member perceives the environment is adequate for innovation (Dunegan et al. 1992). Leader-member exchange relationships were measured using LMX-7 (Graen and Uhl-Bien 1995). The LMX-7 consists of seven items that characterize various aspects of the relationship between supervisor and subordinate, including trust, support and mutual respect. Cronbach’s alpha was 0.74. We have included the *team average LMX* as a control variable that affects team creative environment. We also calculated LMX within-team standard deviation as differences in co-workers relation to the team leader has been acknowledged to affect team environment (Bakar and Sheer 2013).

### Inter-group Agreement and Reliability (Data Aggregation)

If the team average scores for creative team environment, job complexity and task interdependence reflect a shared reality within each group, the scores obtained from individual team members should be similar. This similarity can be measured using the inter-group agreement coefficient (*r*_wg_) and the inter-class coefficient, ICC(1) and ICC (2) (Bliese and Halverson 1998). The average results for *r*_wg_ when using the uniform distribution were 0.93 (confidence interval (0.73, 1.13)) for creative team environment, 0.90 (confidence interval (0.91, 0.99)) for job complexity and 0.78 (confidence interval: (.53, 1.03)) for task interdependence. The mean *r*_wg_ scores that suggested strong within-team agreement (Biemann et al. 2012:73) support the idea that data can be aggregated at the team level. We also calculated the ICC(1) and ICC(2) indexes (following Biemann et al. 2012). The results for ICC (1) were 0.24 for creative team environment, 0.10 for job complexity and 0.14 for task interdependence. These numbers suggest that the scores obtained from variance analysis depend on membership in a specific team (Bliese and Halverson 1998). The reliability of the team-level means was calculated by ICC(2) (Cole et al. 2013). This index measures reliability in terms of group consistency, yielding scores of 0.92 for creative team environment, 0.81 for job complexity and 0.86 for task interdependence. The levels of all of these coefficients satisfy the suggested criteria for data aggregation (Gilson et al. 2005; Schneider et al. 2005), with a reliable result for the aggregation of creative team environment, task interdependence and job complexity variables.

It should be noted that the case of CWX is different to other variables, such as creative team environment, job complexity or task interdependence, as CWX is not really a team-level but a dyad-level variable. Different levels should be taken with care so that they do not lead to further confusion (Kozlowski and Klein 2000). There are no reasons to expect a very high agreement between the perspectives of all co-workers within a team as the quality of the relationships vary across dyads. Consequently, as this is not a team construct, we have calculated the values of interrater agreement and ICC(1), ICC(2) for CWX at the dyad level. The mean inter-group agreement coefficient (*r*_wg_) was 0.83 (confidence interval (.56, 1.10)), and the resulting values of ICC(1) and ICC(2) also satisfied the criteria for data aggregation (being 0.30 and 0.42, respectively).

## Results and Analysis

We used SPSS to obtain the descriptive statistics of the variables involved in the analysis. Table 1 shows the correlations between variables, as well as descriptive statistics such as the mean and standard deviation. The average team co-worker exchange was relatively high, being on average 3.59. When analysing more of the variable CWX in depth, we found that the within-team standard deviation of CWX was on average 0.64 and its correlation to within-team average CWX was non-significant and close to zero (correlation = −0.087), showing that the average quality of the relationships between two co-workers does not depend on the relations that hold between the rest of the co-workers within the team. The correlation between within-team average CWX and the standard deviation of LMX was negative (−0.117), and even if it was not significant, the regression coefficient was found to be significant and negatively related to team creative environment (Table 3), indicating that the higher the differences of the quality of the relationships that the team leader holds with the co-workers, the lower the average of the relationships that co-workers hold between them. The average team LMX was relatively high, around 3.77. The correlation analysis of the key variables initially confirms the hypotheses. Specifically, the CWX variable correlates positively with creative team environment (*r* = 0.476***) and, to a lesser extent, possibly because of the indirect effect, with published papers (*r* = 0.235*); job complexity also correlates positively with creative team environment (*r* = 0.314) though it does not correlate with team performance. As expected, team performance highly correlated with team size and average team member’s tenure.

**Table 1:** Descriptive statistics and correlations

		Mean	Std. dev	1	2	3	4	5	6	7	8	9
1	Average team CWX	3.594	0.500	1								
2	Size	5.642	2.685	0.033	1							
3	Average team tenure	40.444	17.943	0.104	0.137	1						
4	Average team LMX	3.790	0.495	0.167	−0.192	−0.048	1					
5	LMX within-team dispersion	0.493	0.357	−.117	−0.120	−0.035	0.252*	1				
6	Job Complexity	4.003	0.331	0.076	−0.115	−0.049	0.256*	−0.015	1			
7	Interdependence task	3.827	0.522	0.339**	−0.122	0.049	−0.006	−0.122	0.380**	1		
8	Team creative environment	3.852	0.465	0.476**	0.139	0.137	0.141	−0.297**	0.314**	0.502**	1	
9	Team performance	6.827	4.180	0.235*	0.596**	0.282*	0.000	−0.090	0.042	0.103	0.371**	1

***p < 0.001; **p < 0.01; *p < 0.05; †*p* < 0.10

We used path analyses in MPlus 7.4 (Muthén and Muthén 1998–2015) to test the mediating effect of creative team climate and the moderation effect that job complexity and interdependence of the task exert in the relationship between average CWX and team performance. As the dependent variable accounts for the number of published papers, it is a count variable and a normal distribution cannot be assumed. Consequently, we used a Poisson distribution and maximum likelihood estimation with robust standard errors. We then used a bias-corrected bootstrapping method to test for the indirect and mediation effects through robust estimators. Table 2 shows the results of a path analysis to test the simple mediation model. The results provide support to Hypothesis 1 that average CWX relationships relate positively to creative team environment. As can be observed, the coefficient for the variable CWX becomes statistically significant (Table 2, Model 1).

**Table 2:** Results from mediation analysis

	*Model 1*			*Model 2*		
	*DV: Creative team environment*			*DV: Team performance*		
	Beta	SE	*P* value	Beta	SE	*P* value
(Constant)	4.082	1.808	0.024	−2.896	1.590	0.068
Team size	0.114	0.098	0.244	0.696	0.098	0.000
Team average tenure	0.078	0.094	0.407	0.193	0.131	0.141
Team average LMX (LMX)	0.168	0.114	0.139	0.033	0.141	0.813
Team LMX dispersion	−0.276	0.165	0.094	0.124	0.155	0.421
Team average CWX(CWX)	0.404	0.111	0.000	0.265	0.175	0.130
Creative team environment				0.427	0.169	0.012
Chi-squared	−231.500 (15 degrees of freedom)					
	(AIC 493.197; BIC 529.114)					
Indirect effect	0.132 CI (95%): (0.03, 0.37)					
Total effects	0.334 CI (95%): (0.094, 0.547)					

Hypothesis 2 states that creative team environment relates positively to team creative performance, which we measure as number of published papers produced by the team over 5 years, and effectively, the coefficient for creative team environment reaches statistical significance (Model 2 in Table 2), providing support for hypothesis 2.

For hypothesis 3, we first calculated the indirect coefficient which was found significant (*p* value = 0.035). Then we follow the traditional perspective provided by Baron and Kenny (1986) and modified by Preacher and Hayes’s (2008) bootstrapping method. In Model 1 of Table 2, the coefficient of team average CWX must be significant and we can observe that it is significant (so that team average CWX is related to Creative Team Environment); in Model 2 of Table 2, team average CWX is not significant to team creative performance when the variable creative team environment is also in the equation (Model 2 in Table 2). The indirect effect of CWX on team performance is defined as the product of the path *CWX* → *creative team environment* (*a*) and the *creative team environment* → *team performance* path (*b*), or *ab*. Recent literature suggests the use of bootstrapping methods (Preacher and Hayes’s 2008) to test for the significance of the indirect effect parameter. We applied the corrected bias bootstrapping method, which outperforms the usual bootstrapping method. Results showed that the path from average CWX to creative team climate was significant (95% CI = [0.156, 0.617]), suggesting that better co-worker relationships lead to higher creative team climate. Bias-corrected bootstrap confidence intervals also showed that the path from creative team environment to team performance was significant (95% CI = [0.077, 0.679]). Finally, we also found that the bootstrap confident interval for the indirect effect (equal to 0.132, 95% CI = [0.030, 0.378]) was significant. Consequently, we can confirm that our data supports hypothesis 3.

To test hypothesis 4 (that job complexity, interdependence task and average CWX interact in their relation to creative team environment), we conducted hierarchical regression analysis (Models 1 and 2 in Table 3), including as control variable size, tenure, team average LMX and team LMX dispersion in the first step. The results show that the two-way and three-way interaction terms are highly significant at the 5% level which gives support to hypothesis 4. The Akaike’s Information Criterion (AIC) provides an index that can be used to compare models. It is based on the likelihood function and has been proved to perform better than likelihood ratio tests. The smaller value of the AIC index for model 2 in Table 3 also provides support to this hypothesis.

**Table 3:** Results from hierarchical regression analysis (dependent variable: *Creative Team Environment*)

	*Model 1* *DV: Creative team environment*			*Model 2* *DV: Creative team environment*		
	Estimate	SE	*P* value	Estimate	SE	*P* value
(Constant)	0.277	0.563	0.623	−54.504	18.682	0.004
Team size	0.031	0.015	0.033	0.039	0.014	0.005
Team average tenure	0.002	0.002	0.373	0.001	0.002	0.804
Team average LMX (LMX)	0.147	0.085	0.084	0.154	0.079	0.052
Team LMX dispersion	−0.305	0.110	0.006	−0.292	0.107	0.006
Team average CWX(CWX)	0.262	0.083	0.002	16.441	5.262	0.002
Job complexity (JC)	0.201	0.128	0.116	14.226	4.630	0.002
Interdependence task (IT)	0.306	0.084	0.000	15.094	5.001	0.003
CWX × JC				−4.134	1.299	0.001
CWX × IT				−4.301	1.379	0.002
IT × JC				−3.782	1.236	0.002
CWX × IT × JC				1.097	0.339	0.001
Chi-squared	52.729			64.572		
	AIC 70.139; BIC 91.689			AIC 66.296; BIC 97.424		

**Fig. 2:**
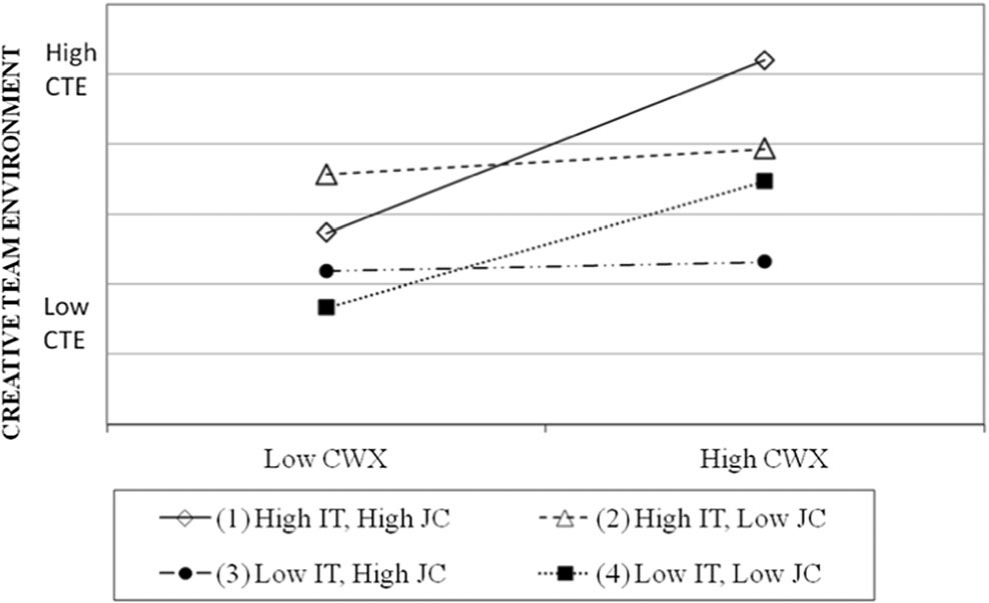
Moderation effect of job complexity (*JC*) and interdependence task (*IT*) in the relationship between team average CWX and Creative Team Environment

Figure 2 plots the moderation effect that job complexity and interdependence task exert on the relationship between team average CWXs and creative team environment at high and low levels of the variables (Aiken and West 1991), defined as one standard deviation above and below the mean. We analysed the slopes throughout the different values of the variables. The slope for teams with high job complexity and high interdependence task was significantly different from zero (*p* value = 0.000). We can then maintain that teams with high job complexity and high interdependence task present high creative team environment as we predicted, and the higher the average CWX, the higher the creative team environment, providing support to our fourth hypothesis. On the contrary, the slope for teams with either high job complexity and low interdependence task or low job complexity and high interdependence task was not significantly different from zero (*p* value = 0.659 and *p* value = 0.6799, respectively), which implies that for these teams, the positive effect of CWX is independent of the effect of the other two variables. We also tested the differences between the slope for the case of high job complexity and high interdependence task and each of the rest of the slopes by adapting the procedure of Dawson and Richter (2006) to our case. They were all significantly different from the slope for the case when job complexity and task interdependence are both high (lowest *p* value = 0.12), suggesting that, as theorized, in this case the slope is higher (more positive) than in the rest of the cases. This is somehow intuitive as if the job is complex, but the team members can work independently of each other or if they need the other team members to complete their task; however, if such task is not complex, the quality of the relations may not be so relevant.

On the other hand, teams with low job complexity, low interdependence task and low quality relationships among team members are the ones that present the lower creative environment, which increases when CWXs increases. In this case, the slope is again statistically not different from zero (*p* value = 0.007). Consequently, we posit that average CWX exerts a positive effect on creative environment (given the positive and significant direct effect), and that this effect is reinforced in cases of high job complexity and high interdependence of the task, which is when high-quality relationships are more needed. On the contrary, the positive effect of team average CWX is not reinforced when job complexity is low and interdependence of the task is high or when job complexity is high and interdependence of the task is low.

Finally, regarding control variables, we can observe that team size is positively related to team performance (Table 2), in line with recent literature on team innovation (Peltokorpi and Hasu 2014) and to team creative environment (Table 3). Team average tenure was found to be non-significant in all models. While LMX appears to be statistically non-significant or weakly significant (10% level), its dispersion seems to be negatively correlated to team creative environment (Table 3).

We also tested the moderated mediation model, which showed that the indirect effect of CWX on team performance was also moderated by job complexity and interdependence of the tasks, being significant for high levels of both, job complexity and interdependence of the task (95% CI [0.023, 0.324]) and for low levels of both variables (95% CI [0.001, 0.252]), being the moderation non-significant for low (high) levels of job complexity and high (low) levels of interdependence of the task.

## Discussion and Conclusions

Literature has often focused either on leader-subordinate relationships or on the team-level supporting environment and team innovation (Peltokorpi and Hasu 2014). However, meta analyses on team outcomes (Hülsheger et al. 2009) found that some team-level climate-related variables possess a null or moderated relationship to team innovation, whereas higher focus on internal communication among team members can be critical for team innovation (Hülsheger et al. 2009).

The literature shows that interaction with others is necessary for the development of individual creativity (Amabile 1988; Woodman et al. 1993). Quality of co-workers’ relations can either enable or prevent a safe and trustworthy environment, which is crucial to productive performance within a team. For our sample, higher levels of team average CWX relationships are positively related to the establishment of a favourable creative team environment. Sherony and Green (2002) did not find a positive relationship between overall CWX quality and work attitudes, and they argued that the relationship might be more powerful if CWX quality was tested specifically for “significant” peers or co-workers representing critical dependencies. Our choice of R&D teams meets this condition, and our results support the idea that, in general, good relationships among peers in a team favour an environment that fosters creativity. Our findings support the hypothesis that team environment mediates the relationship between the quality of CWXs and team performance, extending previous literature on the essential role played by the environment to finally achieve team outputs.

There is evidence of a direct relationship between job complexity and individual benefits and responses (number of new ideas generated, work challenge, learning and personal opportunities; see Hatcher et al. 1989; West 2002); but we focus largely on creative environment and team results. Previous studies indicate that the more complex the task is, the greater is the frequency of interaction needed between team members; thus, it makes sense that the environment is benefited.

Similarly, studies of group behaviour show that the degree of cohesion and quality of interaction between members influence performance and effectiveness within teams (King and Anderson 1995): Teams whose members are closely interrelated are more efficient than those with more independent members. However, this connection is much more complicated than that so that for the different combination of these variables, the quality of CWXs plays a different role, exerting a higher or lower effect in the construction of a creative team environment. From our results, it seems that when job complexity is low but task interdependence is high, the simplicity of the task makes team members in less need of holding particularly good relationships to achieve a creative environment. They need each other because of high task interdependence, but because the task is simple, they need fewer interactions, less cognitive cohesion and fewer discussions so that the quality of the relations they hold is not so relevant. The same occurs in the case of low task interdependence and high task complexity. Complex tasks are challenging and motivating for team members developing them. However, if the task needs low interdependence among team members, they will have to work independently to each other and will need to focus on the complex task in order to be able to complete it, pushing into the background personal relations. Consequently, in this case, CWXs are not crucial to achieve a team creative environment. The opposite occurs when interdependence task is high and job is complex and therefore requires exchanges of ideas. If this is the case, a key element is holding safe and trustworthy relationships among team members, promoting such exchanges. Consequently, if team members need each other to complete their tasks, which are complex and in need of exchanges, co-worker relations are vital. Consequently, hypothesis 4 was supported as for the case of high job complexity, and high interdependence task, team average CWX is strongly positively related to creative team environment. Unexpectedly, CWXs were found to be highly significant in the case of low task complexity and low interdependence task. A potential reason is that the low need for interactions to complete the jobs and the simplicity of the task makes the work less challenging and less motivating so that holding good relationships is the only way to create a positive creative environment.

The results of this study have important practical implications for both team leaders and organizations who wish to encourage and support creative behaviour within teams and among individual members. Our findings show that the creative/innovative output of a team depends not only on the leader but also on relationships among team members. A second practical implication for team leaders and managers could be extracted from our results: Good CWXs generally contribute to creative team environments, but they may be essential where jobs are complex and tasks are designed to be highly interdependent. Furthermore, a third key implication can be derived from the idea of Wageman (1995), who suggested that the level of interdependence necessary to carried out certain tasks may differ, depending on how the tasks have been designed. We can conclude that to promote creative team environments, managers should design tasks in such a way that interdependence among team members is high, particularly when tasks are of high complexity. Finally, this study supports the idea that a proper work environment should be promoted to enhance team performance.

Our study was limited by the particular nature of our sample, scientific and university R&D teams with established innovation intentions and complex tasks. It would be advisable to conduct similar studies in different geographic and industrial contexts in order to ascertain the possible roles of other factors such as culture, level of economic development or resources. Also, this study simply averaged the scores for dyadic relationships within a team, assuming that the more there are high-quality CWXs, the greater would be the team performance, although members do have multiple interactions with different dyads within their teams. It remains for future research to determine the potential interactions between dyads in a team and extend LMX differentiation theory. Specifically, the role played by CWX differentiation in a team needs further study.

We left for future research providing empirical evidence of the theoretical arguments presented in this work in other contexts, particularly in contexts where the power of the leader may be stronger. In our empirical application, R&D teams, the power of the leader can be slightly lower than in other settings. While frequently promotion of team members depends partly on the results and resources provided by research projects that are usually led by team leaders, these team leaders possess a relatively lower power on team members than, for example, team leaders of a private firm. This fact may change the tradeoff between the variables considered. In the case of a more powerful leader, the effect of CWX and LMX may interact to a greater extend so that co-worker relations may be partly driven by the relation that each co-worker holds with the team leader. Still, even in our case, LMX dispersion was negatively correlated to creative team environment, which initially implies that high differences in the relationship that the leader hold with the different co-workers may negatively affect relationships between co-workers. This topic was not the objective of this work, but this is an interesting issue that is in need of further study which we left for future research. Task complexity and interdependence may also play a different role, as situations of lower interdependence and complexity may enhance the importance of personal relations in a setting of very powerful leaders.

Finally, we would also like to point out that, as in any other study, the variables included in the study are limited by nature with respect to what they measure. There are many other facets in co-worker relationships that are not captured by the CWXs items. Furthermore, the addition of other variables as controls such as organizational support and/or organizational climate, as Chiaburu et al. (2013) suggested, that affect team performance would be desirable.
